# Implementation of a standardised annual anticoagulation specialist review in primary care

**DOI:** 10.1017/S1463423620000171

**Published:** 2020-06-09

**Authors:** Nathan W. Hutchinson-Jones, Sophie K. Didcott, Matthew D. Jones, Josephine N. Crowe

**Affiliations:** 1 Anticoagulation Pharmacist, Royal United Hospital, Bath; 2 Anticoagulation Nurse, Royal United Hospital, Bath; 3 Lecturer in Pharmacy Practice, University of Bath; 4 Consultant Haematologist, Royal United Hospital, Bath

**Keywords:** anticoagulation, direct oral anticoagulants, review, service development warfarin

## Abstract

An increasing number of patients are being prescribed direct oral anticoagulants (DOACs), while the patients who remain on warfarin are becoming more complex. There is currently a lack of a standardised anticoagulation review for patients in primary care, resulting in potentially preventable harm events. Our aim was to implement a new service, where a standardised review is carried out by a specialist multidisciplinary secondary care anticoagulation team. Overall, the implementation of a standardised review resulted in better optimisation of anticoagulation management for patients taking either a DOAC or a warfarin. Of the 172 eligible patients prescribed warfarin, 47 (27%) chose to switch a DOAC. The average time in therapeutic range for patients on warfarin before and after the pilot increased from 73.5% to 75%. Of 482 patients taking a DOAC, 35 (7%) were found to be on incorrect dose. In 32 (91%) of 35 patients, the dose was amended after notifying the patient’s general practitioner. We also found a significant number of patients inappropriately prescribed concomitant medication such as antiplatelet or non-steroidal anti-inflammatory drugs, potentially putting the patients at an elevated risk of bleeding. While further research is needed; we believe the results of this pilot can be used to help build a case to influence the commissioning of anticoagulation services. Secondary care anticoagulation teams, like our own, may be well-placed to provide or support such services, by working across the primary care and secondary care interface to support our primary care colleagues.

Oral anticoagulants are high-risk medicines associated with significant harm events, especially if not managed appropriately (Howard *et al*., 2007; Maffey *et al*., 2018). Two major classes of oral anticoagulants are available: vitamin K antagonists (typically warfarin) and direct oral anticoagulants (DOACs). Over the past five years, our hospital has established a specialist anticoagulation team responsible for continuously improving the care of patients taking these medicines. As part of this programme or work, we piloted a standardised annual anticoagulation review for patients in primary care. The rationale for this service, and its implementation and outcomes are described in this article.

To provide context, our hospital has 759 beds and provides comprehensive acute, outpatient and diagnostic services to a population of ~500,000 people in towns and villages in a predominately rural area of south-west England. In our local area, responsibility for the routine prescribing and monitoring of the DOACs lies with general practitioners (GPs). Warfarin is also prescribed by GPs, but the responsibility for warfarin monitoring varies with GP practice and may be provided by either the GP or the hospital anticoagulation team. Eleven of 26 GP practices in our local area currently subcontract their warfarin monitoring to the hospital anticoagulation team.

## Scoping audit: the extent of anticoagulation-related harm at hospital admission

From January to December 2016, we carried out an audit of patients admitted to hospital with known atrial fibrillation (AF) who suffered an anticoagulation-related harm event and patients prescribed warfarin (for any indication) who were admitted due to a high international normalised ratio (INR) of greater than eight. Over this 12-month period, 201 patients met the criteria for inclusion in the audit. In all, 135 admissions (67%) were patients with known AF (Table 1). In 57% of these patients there was a problem with their anticoagulation which might have been detected before admission, such as a sub-therapeutic INR, prescription of an incorrect DOAC dose or non-adherence. In all, 68 cases (34%) has an INR of greater than eight was found to be a contributory cause for a patient’s hospital admission. Two of these patients had known AF and were admitted with a central nervous system bleed (Table 1). The mean length of stay for these patients was 14 days. Overall, anticoagulation-related harm events resulted in a total of 1575 bed days, with a mean length of stay of 29 days. The mortality rate was 20% within four weeks of admission. Our audit did not include patients who were on anticoagulation for other indications such as treatment and prevention of venous thromboembolism (VTE). Consequently, our data are likely to be an underestimate of the true extent of anticoagulation-related harm events. Overall, the results suggest identifying anticoagulation problems in primary care can help reduce patient harm and demand for secondary care services.

**Table 1.** Results of an audit of patients with known atrial fibrillation admitted to hospital with an anticoagulation-related harm event from January to December 2016.

		Patients prescribed warfarin		Patients prescribed a DOAC		
	Total number of patients (%) (*n* = 135)	Total number of patients	Number of patients with different levels of INR control	Total number of patients	Number of patients with different types of problem	Unknown anticoagulant
Admitted with an ischaemic stroke while not anticoagulated	54 (40%)	-	-	-	-	-
Admitted with an ischaemic stroke while anticoagulated	47 (35%)	28	Sub-therapeutic INR: 17	19	Prescribed incorrect lower dose: 3	0
					Not taking prescribed DOAC: 3	
			Therapeutic INR: 11		Not taking rivaroxaban with food: 2	
					No known problem: 11	
Admitted with a central nervous system bleed while anticoagulated	30 (22%)	11	INR >8: 2	11	Prescribed incorrect higher dose: 6	8
			INR >5: 6		No known problem: 5	
Admitted with another or unspecified anticoagulation-related harm event	4 (3%)	3	-	1	-	-

DOAC = direct oral anticoagulant; INR = international normalised ratio

## Addressing the problem identified by the audit

Since our audit, patients with known AF, not currently anticoagulated, are reviewed annually in primary care by GP practice pharmacists. However, the problem of safe ongoing management of patients already established on long-term anticoagulation has not been addressed. The London Clinical Network published guidance in 2016, which defined the elements of an ‘excellent’ anticoagulation service (Kar and Williams, 2016). This was primarily for patients anticoagulated for AF; however, the general principles are transferable to patients on oral anticoagulation for other indications too. An annual anticoagulation review is classed as an essential element for providing an excellent service. Other guidelines recommend the use of a multidisciplinary team working to standardised policies to improve anticoagulant prescribing and the development of a shared care approach across the interface between primary and secondary care (Kvamme *et al.,* 2001; Barr and Epps, 2019). Currently there is no standardised review for patients prescribed a DOAC or warfarin (regardless of indication) in our local area or nationally. There is also little guidance on how best such a review might be implemented. Therefore, in order to address the problems identified by our audit, we decided to develop and pilot a new service whereby a specialist multidisciplinary secondary care anticoagulation team provided a standardised annual anticoagulation review for patients in primary care. The aim of this service was to optimise anticoagulant treatment and thus reduce the risk of harm.

## Implementing an annual anticoagulation review

The annual anticoagulation reviews were designed to include content specified by the London Clinical Network (Kar and Williams, 2016). They were carried out by a specialist nurse and pharmacist, both with several years’ experience of working in anticoagulation and employed by our hospital. A review was carried out for all patients prescribed an oral anticoagulant (warfarin or DOAC) in five GP practices to which the hospital anticoagulation team currently provides a warfarin monitoring service. These five GP practices had a total of 32,024 registered patients and 29 GPs. Reviews were carried out in 2018 over a 12-month period. In order to provide a service which is as close and convenient as possible for patients (Kar and Williams, 2016), all reviews were carried out either at their GP practice or via telephone.

### Warfarin reviews

Where possible, warfarin reviews were carried out either face-to-face at the patient’s GP practice or via a telephone consultation. In all cases, the consultation was recorded on the patient’s GP record and, if necessary, a recommendation was made to the GP for follow-up. Where a patient was not able to be contacted, a more limited review was carried out in the patient’s absence and recommendations for follow-up, if applicable, were made to the patient’s GP. For all patients taking warfarin, the following criteria were reviewed:

1. Indication
2. Assessment and correction (if possible) of risk factors for bleeding (eg, concomitant prescribing of antiplatelet and or non-steroidal anti-inflammatory drugs (NSAIDs)
3. Time in therapeutic range (TTR; with a target of >65%)
4. Alternative anticoagulation: appropriate patients were given the option of all four DOACs as alternative anticoagulants as part of a joint discussion

For patients who were reviewed either face-to-face or via a telephone consultation, the following criteria were also reviewed:

1. Patient’s understanding of his/her condition and treatment
2. Adherence
3. Possible side effects

Patients prescribed warfarin for AF associated with valve disease (as per the European Society of Cardiology guidelines, 2018), mechanical prosthetic valve replacement or previous VTE with an INR target >2–3 were deemed inappropriate for a switch to a DOAC (Steffel *et al*., 2018). In addition, patients with a weight of >120 kg were ineligible for a switch to a DOAC (Martin *et al*., 2016). Patients were also deemed inappropriate for a DOAC if they had any other contraindication as per the relevant summary of product characteristics (SPC). For all patients who continued with warfarin, advice regarding improving INR control was given. For appropriate patients who decided to switch to a DOAC, the GP was contacted, so that a prescription for the DOAC could be issued based on the recommendation of the anticoagulation nurse or pharmacist. A switching plan was then given to the patient (Supplementary File 1). Individual switching plans for each DOAC were created by the hospital anticoagulation team using information from the SPCs of the various drugs. Patients were given the contact details of the hospital anticoagulation team in case they had any questions or concerns after switching.

### DOAC reviews

DOAC reviews were completed in two parts. Part one involved a review of the patient’s GP record was done in the absence of the patient and included the following criteria:

1. Indication
2. Assessment and correction (if possible) of risk factors for bleeding (eg, concomitant prescribing of antiplatelet and or NSAIDs)
3. Dose
4. Renal function, liver function, and full blood count (in the last 12 months)
5. Weight

For part two, the following criteria were assessed via a telephone consultation with the patient:

1. Patient’s understanding of their condition and treatment
2. Adherence
3. Alternative anticoagulant strategies (if applicable)
4. Taking with food (if prescribed rivaroxaban)
5. Missed doses (including the number of missed doses and any reasons why)
6. Possible side effects

All complex patients (taking warfarin or a DOAC) were discussed as part of a multidisciplinary meeting involving a consultant haematologist and other specialist as applicable (Kar and Williams, 2016). If necessary, patients were referred for review at the haematology thrombosis clinic.

## Outcomes

Over a 12-month period, 821 patients were reviewed as part of the pilot. Of these patients, 339 (41%) were initially taking warfarin and 482 (59%) taking a DOAC.

### Warfarin reviews

Warfarin reviews were carried out either face-to-face with the patient at the patient’s GP practice (*n*=162; 48%) via a telephone consultation with the patient (*n*=53; 16%) or in the absence of the patient (*n*=124; 37%). Patients who were reviewed in their absence included those patients under the care of a nursing or residential home and those who were uncontactable via telephone or letter. Patients had been prescribed warfarin for a variety of indications (Table 2).

**Table 2.** Indications for which reviewed patients had been prescribed warfarin

Indication	Number of patients
Non-valvular atrial fibrillation	223
AF associated with valve disease^a^/mechanical prosthetic valve replacement	33
Cardiomyopathy	2
Previous VTE	73
Previous VTE with higher INR target^b^	8
**Total**	339

a Valve disease was defined as per the guidance from the European Society of Cardiology (ESC); moderate to severe mitral stenosis or rheumatic mitral stenosis

b A higher INR target was defined as any INR target >2–3 (eg, 2.5–3.5)

AF = atrial fibrillation; VTE = venous thromboembolism; INR = international normalised ratio

Of the 339 patients taking warfarin, 228 (67%) were deemed eligible for a switch to a DOAC. Of these patients, 56 (25%) had a review done in their absence. Instead a recommendation was made to their GP for follow-up . Of the remaining 172 patients, 47 (27%) chose to undertake a switch to a DOAC. Reasons for patients switching included one or more of the following:

1. TTR <65% (19 (40%) of 47 patients who switched to a DOAC had a TTR <65%. Of the 19 patients, 4 (21%) had a TTR <40%)
2. Lack of regular INR monitoring
3. Fewer interactions between warfarin and diet/medication
4. Lower risk of bleeding

Reasons given by patients for not switching included one or more of the following:

1. TTR > 65%
2. Lack of reversal agent
3. Previously not tolerated a DOAC
4. Enjoyed the contact with a healthcare professional at the INR clinic
5. Wished to discuss with their usual GP, healthcare professional or family

All appropriate patients were given the option of all four DOACs as alternative anticoagulation. Of the 47 patients who switched to a DOAC, 22 (47%) chose apixaban, 14 (30%) chose edoxaban and 11 (23%) chose rivaroxaban. Factors that affected their choice included:

1. Dosing frequency
2. Bleeding risk
3. Need to take with food
4. Familiarity with the chosen DOAC

Anticoagulation was stopped completely in two patients (0.6%) after being reviewed at the haematology thrombosis clinic. Each patient who was reviewed by a face-to-face or telephone consultation was asked whether they understood why they were prescribed warfarin (*n*=215). Most patients knew why there were taking warfarin, even if this was only a basic understanding (eg, thins blood and prevents clots). A small number of patients (*n*=5; 2.3%) did not know why they were taking warfarin. Only four patients (2%) reported the possible side effects, which included bruising, headaches and aching. All patients were referred to their GP for advice. At baseline, there were 76 patients (22%) with a TTR of <65%, which is associated with an increased risk of stroke and bleeding (Jones *et al*., 2005). The average TTR for all five GP surgeries before and after the pilot increased from 73.5% to 75% (based on the INR data six months prior to commencing the pilot and six months after starting). Concomitant prescribing of antiplatelet or NSAID was found in seven (2%) patients taking warfarin. Unless there was a clear reason documented in the patient’s notes as to why he/she was on this combination, a task was sent to the patient’s GP to review. Of the seven patients, two (29%) had their antiplatelet or NSAID stopped.

### DOAC reviews

A total of 482 reviews were carried out without the patient using their GP record. Tables 3 and 4 summarise the DOACs prescribed to these patients and their indications.

**Table 3.** DOAC initially prescribed for reviewed patients

DOAC	Number of patients
Apixaban	300 (62%)
Dabigatran	19 (4%)
Edoxaban	3 (1%)
Rivaroxaban	160 (33%)
**Total**	482

DOAC = direct oral anticoagulant

**Table 4.** Indication for a DOAC in reviewed patients

Indication	No. of patients
NVAF	374 (78%)
Previous stroke or transient ischaemic attack	8 (2%)
Other cardiology	3 (1%)
Previous VTE	95 (20%)
Arterial thrombosis	2 (0.4%)
**Total**	482

NVAF = non-valvular atrial fibrillation; VTE = venous thromboembolism

Of the 482 patients taking a DOAC, 35 (7%) were found to be prescribed a potentially incorrect dose (31 patients were prescribed apixaban, 2 were prescribed dabigatran and two were prescribed rivaroxaban). Of the 35 patients, 28 (80%) were prescribed too small a dose. Twenty-six of these patients were prescribed the lower dose of apixaban (2.5 mg twice daily). In 32 (91%) of these 35 patients, the dose was amended after notifying the patient’s GP. Of the three patients who did not have their dose changed, one had their apixaban stopped, the second one was on the lower dose due to prostate cancer (on the advice of a consultant oncologist) and the third one was on the lower dose as they were also prescribed aspirin (on the advice of consultant cardiologist). Concomitant prescribing of an antiplatelet or NSAID was found in 39 (8%) patients taking a DOAC. Unless there was a clear reason documented in the patient’s notes as to why they were on this combination, a task was sent to the patient’s GP to review. Of the 39 patients, 4 (10%) had their antiplatelet or NSAID stopped. Of the 482 patients taking a DOAC, 71 (15%) had not had any blood tests in the previous 12 months. In each case, the GP was notified and asked to request for and check the blood test reports. Of the 482 DOAC patients, 126 (26%) went on to have a review via a telephone consultation. Reasons for not carrying out a telephone review included:

1. Patient under the care of a residential or nursing home
2. Unable to contact the patient
3. Time running out prior to the completion of the project

For patients taking rivaroxaban, 3 (7%) of 46 were found not to be taking their anticoagulant with food, which significantly reduces its bioavailability (Stampfuss *et al*., 2013). We did not find any patients who were completely non-compliant with taking their DOAC; however, one patient was found to be taking apixaban once daily rather than twice daily. Potential side effects were described in 8 (6%) of 126 patients, including bleeding gums, dizziness, nose bleeds and lethargy. In each instance, the patients were advised to speak to their GP. Four (3%) of 126 patients who had a telephone consultation did not know why they were taking their DOAC.

## Potential effectiveness of the annual review service

Overall, the results of our pilot suggest that a standardised annual anticoagulation review completed in primary care by a specialist multidisciplinary secondary care anticoagulation team is feasible. Numerous problems were identified (such as TTR <65%, incorrect DOAC doses and non-adherence), giving the opportunity to reduce anticoagulation-related harm events by optimising anticoagulation therapy.

Of the patients who switched from warfarin to a DOAC, 49% had a TTR <65%, which is associated with an increased risk of stroke and bleeding (Jones *et al*., 2005). This risk is likely to have been reduced by the change in these patients’ medication. For patients who remained on warfarin post-review (regardless of whether they were eligible for a switch to a DOAC or not), there was an improvement in TTR from 73.5% to 75%. This was most likely because the patients were receiving advice on how to improve their INR control, for example, via a consistent diet and good adherence.

We found that only 27% of patients taking warfarin who were eligible for a switch to a DOAC chose to do so, which is a similar proportion to previous research (Ikeda *et al*., 2018). Some of these patients were well controlled with a TTR of >65% and were happy to continue with warfarin; however, some with a TTR of <65% were still reluctant to switch. Patients’ reasons for this included concern over a lack of a reversal agent and lack of contact with a healthcare professional during INR checks, both of which were also reported in a recent systematic review (Wilke et al., 2017). Wider availability of reversal agents may encourage more patients to switch to a DOAC in the future and thus increase the potential impact of a specialist anticoagulation review (Pollack *et al.,* 2015; Galliazzo *et al.,* 2018; Heo, 2018). There were several patients who initially decided not to change from warfarin to a DOAC, who subsequently decided to switch after the completion of the pilot. For some patients, it was important to them that they discussed this decision with their usual GP, healthcare professional or family. Similarly, it has previously been suggested that lower levels of patient trust in a physician might be associated with poor adherence to oral anticoagulants (Di Minno *et al.,* 2014). This highlights the importance of regular re-assessment of people taking oral anticoagulants but also suggests a potential limitation of such reviews being carried out by an anticoagulation specialist, as some patients preferred to decide with their usual clinician.

In our pilot, 7% of patients already anticoagulated with a DOAC was prescribed the incorrect dose, putting them at an increased risk of thrombosis or bleeding (Dillinger *et al*., 2018). In our local area, all the DOACs are available as options for stroke prevention in non-valvular atrial fibrillation and treatment and prevention of VTE; however, apixaban is a popular choice based on a good bleeding risk profile versus warfarin and a lack of initial bridging therapy with a low-molecular-weight heparin (Vinogradova *et al*., 2018). Apixaban dosing is dependent on renal function, age and weight (Bristol-Myers Squibb-Pfizer, 2019). All these factors may change with time and consequently result in patients being prescribed an inappropriate dose. In addition, 15% of patients taking a DOAC had not had any blood test monitoring within the last 12 months, which does not follow the good practice guidance and makes it difficult to assess the appropriateness of their DOAC dose (Conway *et al*., 2017; Specialist Pharmacy Service, 2019). Our annual anticoagulation review service therefore helped to ensure patients were prescribed the correct dose on a long-term basis, thus increasing the safety of their treatment.

Concomitant prescribing of antiplatelet or NSAIDs was found in some patients on DOACs and warfarin. This is associated with a significant increased risk of bleeding (Hansen *et al*., 2010; Cannon *et al*., 2017; Janardan and Gibbs 2018), which our annual review service was able to identify and correct if appropriate. Decision-making in primary care on whether to stop an antiplatelet can be difficult, especially as many patients who may require dual antiplatelet and anticoagulant therapy have multiple co-morbidities putting them at a high risk of thrombosis as well as bleeding (Floyd and Ferro 2017). This again highlights the benefit of an annual review by a specialist team with the training, experience and resources to make these decisions.

Rivaroxaban doses greater than 10 mg need to be taken with food to ensure adequate absorption of the drug (Stampfuss *et al*., 2013). Our pilot found that 7% of rivaroxaban patients (all of whom were prescribed 20 mg) were not doing so. Taking higher doses of rivaroxaban without food may increase the risk of thrombosis, so identification of this problem can make a significant contribution to optimising the care of these patients (Schulman, 2017).

The average length of time for each anticoagulation review was 25 minutes. For 821 patients this equated to a total of 342 hours. The estimated total cost per hour of patient contact for a band six hospital nurse is £111 (Curtis and Burns, 2018), which is equivalent to £46.25 per 25-minute review (excluding travel costs). In comparison, the average societal cost of a stroke per patient is estimated to be £45,409 over the first 12 months (Patel *et al*., 2018). More work is needed to evaluate the cost-effectiveness of an annual anticoagulation review; however, the cost of a review appears low in comparison to the cost of an adverse event such as a stroke.

These findings are especially relevant for the context of the United Kingdom’s National Health Service. However, as the health systems of many countries relies on the cooperation of primary and secondary healthcare professionals, we believe this approach might also be useful in other settings, after appropriate adaptations to the local context.

## Challenges and limitations

There were several challenges faced during the project including agreeing participation by the GP practices. Initially this was due to concerns about extra workload for the practice. However, we were able to complete almost all tasks ourselves. One limitation was that we required the patient’s GP to issue a prescription for the DOAC when a switch was agreed, as at the time our anticoagulation nurse and pharmacist were not trained as independent prescribers. Another concern raised by GP practices was the increased cost of prescribing DOACs compared with warfarin. Though drug costs are more expensive, if used effectively DOACs are as cost-effective as warfarin once other costs (such as monitoring) are considered (López-López et al., 2017). This again highlights another reason to ensure patients are optimally anticoagulated with DOACs. Despite the initial concerns of GP practices, feedback from GPs after completing the project was very positive and a high proportion of our recommendations (87%) was put in to action. Subsequently, GPs frequently use the anticoagulation team as a point of contact for relevant queries. Although we did not systematically collect feedback from patients, other studies have suggested that people taking oral anticoagulants value long-term management by anticoagulation specialists (Bartoli-Abdou *et al*., 2018).

Warfarin monitoring services locally are provided by either the hospital anticoagulation team or the GP. All our warfarin reviews were carried out on patients looked after by the hospital anticoagulation team. Consequently, our results may not be reflective of all warfarin patients in the area.

A significant number of reviews were done in the absence of patients, resulting in a more limited review. This was in part due to being unable to contact patients during the limited time at each practice or the patient being at a residential or nursing home. GPs or other healthcare professionals based in primary care may be in a better place to review some of these patients.

Other limitations to our project included not being able to complete all telephone consultations for our DOAC reviews. This was due to the limited funding to complete the project and the significant number of people taking DOACs. Our recommendations were not put into action by GPs in some cases because of the additional clinical information or recommendations which were not available to the specialist review team. This highlights the difficulty in reviewing unfamiliar patients in an unfamiliar setting. Another limitation is that at the start of the project the International Society of Thrombosis and Haemostasis recommended caution with the use of DOACs for patients with a weight of >120 kg (Martin *et al*., 2016). Since then, however, there has been increasing evidence to suggest that the DOACs are safe to use in patients with weights >120 kg (Choi *et al*., 2017).

No routine follow-up was provided for patients who choose to switch from warfarin to a DOAC due to the limited resources to complete the project. However, patients were able to contact the anticoagulation team for advice if needed. Utilisation of community pharmacy services may benefit these patients. The New Medicines Service is an NHS community pharmacy service that provides support to patients with long-term condition’s newly prescribed medication (Elliot *et al*., 2016). Evidence has shown that this service improves adherence to medication (Elliot *et al*., 2014).

Our results do not represent a formally planned evaluation of our new service, so further research is required to fully assess its impact. Ideally, this should include assessment of hard outcomes such as thrombosis and bleeding, patient perspectives and cost-effectiveness.

The London Clinical Networks suggest that many components are involved in the ideal anticoagulation service (Kar and Williams, 2016). An annual review is just one of these components. However, further work is needed at a local level to address other elements of these guidelines in order to improve patient care.

## Conclusions

There is a need to include an annual DOAC and warfarin review in local anticoagulation commissioning and to standardise how patients are managed both locally and nationally. This is especially important as in the future it is likely that the number of patients on DOACs will increase while patients remaining on warfarin are likely to be more complex. Many secondary care anticoagulation teams currently provide a warfarin monitoring service. As the number of people taking warfarin decreases, the role of these teams will need to change. Our pilot suggests that secondary care anticoagulation teams may be well-placed to provide this service, by working across the primary and secondary care interface to support primary care colleagues. Alternatively, GPs and other healthcare professionals in primary care might carry out such a service with training and support provided by a specialist secondary care team.

## Supplementary material

For supplementary material accompanying this paper visit http://dx.doi.org/10.1017/S1463423620000171. click here to view supplementary material
