# Supplementary material for: Implementation of a standardised annual anticoagulation specialist review in primary care
Source: Prim Health Care Res Dev. 2020 Jun 9;21:e17. doi: 10.1017/S1463423620000171 (PMC7303795; doi:10.1017/S1463423620000171)
Supplement: Supplementary file 1 [file S1463423620000171sup001.docx]

**Supplementary File 1**

**Example DOAC to warfarin switching letter**

Date

| Patient Name:  Date of Birth:  MRN:  NHS number: |
| --- |

Dear NAME,

**This plan is for patients who are being switched from warfarin to apixaban**

Apixaban is an example of a Direct Oral Anticoagulant (you may hear them referred to as DOACs). These are an alternative group of drugs to warfarin, they are usually used for:

- Stroke prevention in Non-Valvular Atrial Fibrillation
- Treatment and prevention of blood clots

Apixaban is also occasionally used for other indications.

**Advantages *vs*. disadvantages of taking apixaban instead of warfarin**

| **Advantages of taking apixaban** | **Disadvantages of taking apixaban** |
| --- | --- |
| No common food/drink interactions | No reversal agent – However, the half-life of the drug, which is the time it takes for the amount of drug in your blood stream to reduce by half, is much shorter. Furthermore, the risk of major life threatening bleeding is lower with apixaban compared to warfarin. In addition a reversal agent is also currently in development. |
| No frequent monitoring (no INR monitoring required).  Most patients will have 6 - 12 monthly reviews to check liver function, full blood count and kidney function |  |
| Fewer drug-drug interactions |  |
| Lower risk of life threatening bleeding | Patients with renal disease may need more frequent blood testing. |
| No frequent dose changes required |  |
| Apixaban works quickly once taken | Lack of regular monitoring (some patients may find regular testing re-assuring that warfarin is working and enjoy the contact with a healthcare professional). |
|  | Apixaban needs to be taken twice a day rather than once a day. |

**Apixaban: Key Facts**

- Taken twice daily at either a 5mg or 2.5mg dose depending on renal function, weight and age (you will be advised on the most appropriate dose to take by the anticoagulation team).
- Can be put into a dossette box. If a dose is missed, take your dose immediately and then continue with twice daily intake as before.
- For breastfeeding patients, it is unknown whether apixaban is excreted in human milk, hence apixaban should be avoided. Apixaban should also be avoided in pregnancy. If you become pregnant and are taking apixaban contact a healthcare professional immediately for advice.

More detailed information can be found in the patient information leaflet including common side effects. Contact the anticoagulation team or your GP if you have any concerns.

**The Switching Plan**

Your GP will issue a prescription for apixaban. The following plan should only be started after confirmation from the anticoagulation team. Until then you should cont. to take your warfarin as per normal and you should **not** start apixaban until told to do so.

| **Day 1** | **Last dose** of warfarin |
| --- | --- |
| **Day 2** | **Stop** warfarin |
| **Day 3** | - |
| **Day 4** | INR check   - If INR is **less than 2.0** you can start apixaban.   If INR is too high to start apixaban, book an INR test for 1- 2 days’ time (discuss with anticoagulation team). |
